# Characteristics of coronary artery disease in patients with subclinical hypothyroidism: evaluation using coronary artery computed tomography angiography

**DOI:** 10.1186/s12872-021-02116-0

**Published:** 2021-06-15

**Authors:** Xin-zhu Zhou, Rui Shi, Jin Wang, Ke Shi, Xi Liu, Yuan Li, Yue Gao, Ying-kun Guo, Zhi-gang Yang

**Affiliations:** 1 Department of Radiology, West China Hospital, Sichuan University, No. 37 Guo Xue Xiang, Chengdu, 610041 Sichuan China; 2 Department of Radiology, Key Laboratory of Birth Defects and Related Diseases of Women and Children of Ministry of Education, West China Second University Hospital, Sichuan University, Chengdu, China

**Keywords:** Coronary artery disease, Subclinical hypothyroidism, Coronary artery computed tomography, Coronary plaque subtype

## Abstract

**Background:**

Subclinical hypothyroidism (SCH) has recently been acknowledged as an independent risk factor for coronary artery disease (CAD). However, the characteristics of CAD in patients with SCH are not fully understood. This study aims to evaluate the features of CAD in patients with SCH using coronary computed tomographic angiography (CCTA).

**Materials and methods:**

From 1 April, 2018 to 30 June, 2020, 234 consecutive SCH patients with coronary plaques identified on CCTA were included retrospectively. They were further subdivided into different degree of SCH groups (mild SCH vs. moderate SCH vs. severe SCH: 143 vs 62 vs 28) and different gender groups (men with SCH vs. women with SCH:116 vs 118). The distributions and types of plaques, luminal narrowing, segment involvement scores (SIS) and segment stenosis scores (SSS) were evaluated and compared among the different groups.

**Results:**

Patients with severe SCH had fewer calcified plaques (0.7 ± 0.9 vs. 2.0 ± 1.9, *p* < 0.001) and more non-calcified plaques (0.9 ± 1.0 vs. 0.3 ± 0.5, *p* < 0.001) than those with mild SCH. As the SCH condition worsened, the proportion of non-calcified plaques significantly increased. Whereas there were no significant discrepancies in SIS and SSS among patients with different grades of SCH (all *p* > 0.05). Men with SCH had higher SIS (3.9 ± 2.3 vs. 3.0 ± 2.3, *p* = 0.004) and SSS (7.8 ± 5.4 vs. 5.4 ± 3.0, *p* = 0.002) than women. Multivariate logistic and linear regression analysis demonstrated that grades of SCH (Moderate SCH, odds ratio [OR] 2.11; 95% CI 1.03–4.34, *p* = 0.042; severe SCH, OR: 10.00; 95% CI 3.82–26.20, *p* < 0.001, taken mild SCH as a reference) was independently associated with the presence of non-calcified plaques, whereas sex (B: 1.67; 95% CI 0.27–3.10, *p* = 0.009) was independently associated with SSS.

**Conclusions:**

Severe SCH is associated with non-calcified plaques, and men with SCH have higher total plaque burden than women. We suggest that it is important to evaluate for coronary plaque in SCH patients, especially those with severe SCH and men with SCH.

## Introduction

Coronary artery disease (CAD) remains a leading cause of mortality worldwide and poses a great socioeconomic burden [1]. Classical risk factors of CAD include diabetes mellitus (DM), hypertension, smoking, and hyperlipidemia. In addition, chronic endocrine and metabolic diseases, such as hyperthyroidism and hypothyroidism, also play a key role in the occurrence and progression of atherosclerotic plaques [2, 3].

Subclinical hypothyroidism (SCH) is an early, mild form of hypothyroidism, which is defined as elevated of thyroid-stimulating hormone (TSH) with a normal level of free thyroxine (FT4) level [4]. The incidence of SCH in the iodine-sufficient population is about 10% and increases with age. The incidence rate of SCH is higher in women than men [4]. In patients with SCH,, the sex difference in SCH patients is related to obesity, nonalcoholic fatty liver disease, and impaired endothelial function [5–7].

In contrast to patients with overt hypothyroidism, patients with SCH typically have no clinical symptoms. Therefore, although SCH is a common health problem, it is often overlooked in the study population. Subclinical and overt hypothyroidism are associated with atherosclerosis and CAD [2, 8, 9]. SCH is associated with higher risks of CAD and cardiac mortality, especially in patients with a serum TSH level ≥ 10 mU/L [10]. However, the characteristics of coronary artery atherosclerotic plaques in patients with SCH are not yet fully understood.

The main purpose of this study is to investigate the characteristics of coronary artery atherosclerotic plaques among SCH patients according to the severity of SCH and different sexes using coronary computed tomographic angiography (CCTA).

## Materials and methods

### Study population

From 1 April, 2018 to 30 June 2020, 293 patients with SCH underwent coronary CTA for chest pain or discomfort (51%), palpitation (16%), dyspnea (11%), syncope (1%) and asymptomatic patients for high risk population of CAD (patients with hypertension, hyperlipidemia, diabetes or other risk factors but no obvious clinical symptoms or confirmed coronary artery disease) (21%) in our hospital. Among these patients, 272 patients with coronary artery plaques and had complete clinical and laboratory data were included in this study. The exclusion criteria were the poor quality of images that could not meet the requirement of analysis (n = 7), history of stenting or bypass surgery (n = 23), thyroid hormone replacement therapy (n = 6) or non-ischemic cardiomyopathy (n = 2). Finally, 234 patients (mean age: 71.5 ± 9.8, 116 men (49.6%)) were included in this study. SCH was defined in accordance with The Endocrine Society diagnosis, as the serum TSH ≥ 4.5 mU/L and FT4 within the normal range [11]. The grades of SCH were categorized as mild SCH when TSH level 4.5–7 mU/L, moderate SCH when TSH level 7–10 mU/L and severe SCH when TSH ≥ 10 mU/L [4, 12, 13]. Included patients were further subdivided into different degree of SCH groups (mild SCH vs moderate SCH vs. severe SCH: 143 vs 62 vs 28) and different gender groups (men with SCH vs. women with SCH:116 vs 118). The clinical data and laboratory results were obtained through medical records and patient questionnaires.

### CT scanning protocols

CCTA was performed using a 256 multidetector CT scanner (Revolution CT, GE Healthcare, Waukesha, WI USA). For heart rates of most patients could meet the needs of examination (< 130 beats per minute), beta-blocker preparation was not used for reducing the heart rate. The scanning scope was from the tracheal bifurcation to 20 mm below the inferior cardiac apex. A 70–90 mL (dependent on the body mass index) bolus of iodinated contrast agent (iopamidol, 370 mg of iodine/mL; Bracco Sine Pharmaceutical Corp. Ltd, Shanghai, China) was injected into the antecubital vein at a flow rate of 5 mL/s. Next, a 20-mL saline chaser was injected at the same rate. For scanning parameters, the tube voltage and tube current were set automatically by kV Assist and Smart-mA based on the scout image of the patients, other imagine parameters were 256 × 0.625 mm collimation and 0.28 s gantry rotation time [14]. Retrospective electrocardiographic gating was used to eliminate cardiac motion artifacts.

### Coronary CTA analysis

Acquired data were reconstructed, and a group of images with optimal quality was transferred to a workstation (AW VolumeShare5, GE Healthcare, Waukesha, WI USA) for image analysis. Coronary artery plaques were evaluated through maximum intensity projections, multiplanar reconstructions, curvature plane reconstructions, and volume rendering.

**Fig. 1:**
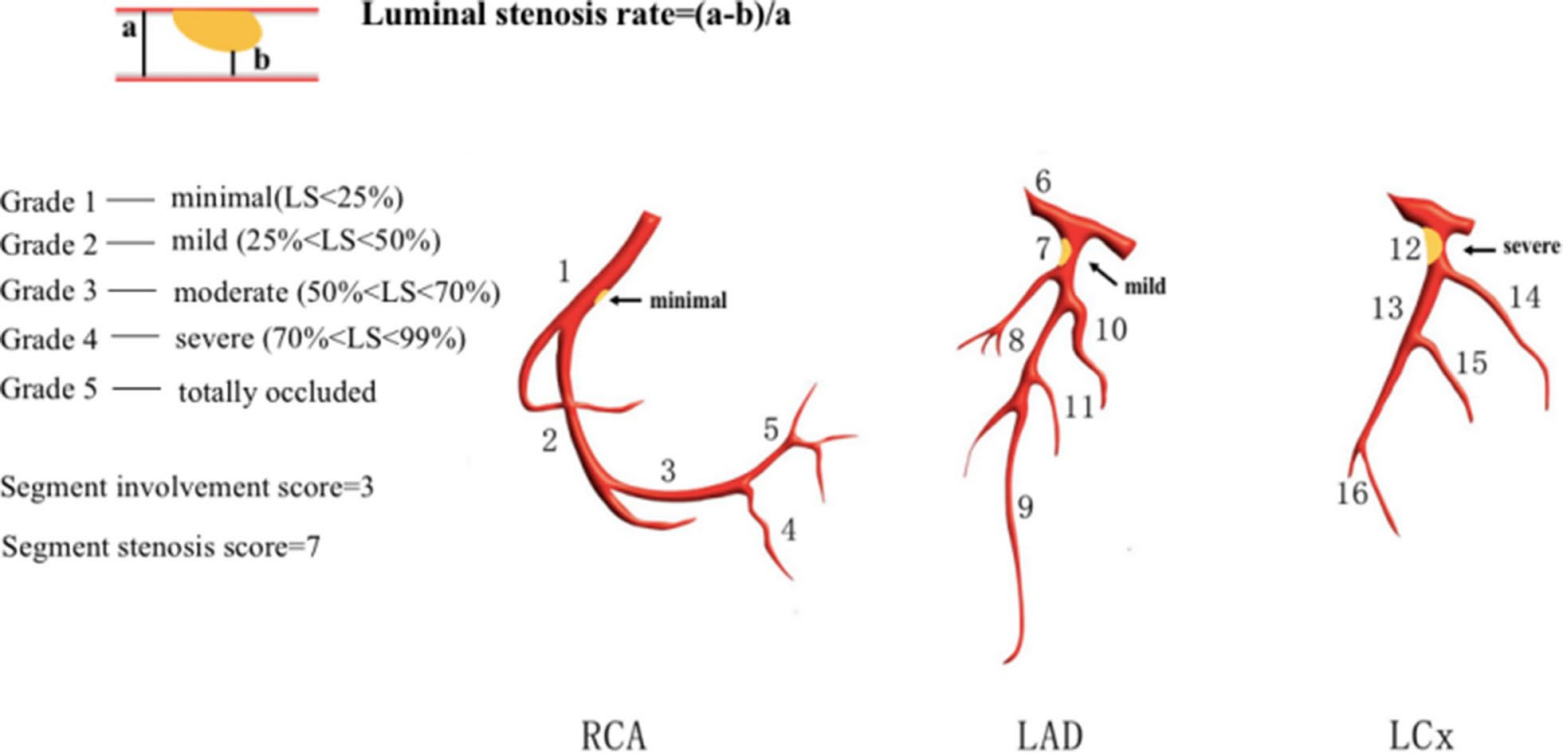
Coronary artery segments: (1) proximal segment of right coronary artery (RCA); (2) middle segment of RCA; (3) distal segment of RCA; (4) right posterolateral artery; (5) posterior descending artery; (6) left main coronary artery; (7) proximal segment of left anterior descending artery (LAD); (8) middle segment of LAD; (9) distal segment of LAD; 10.first diagonal branch; (11) second diagonal branch; (12) proximal segment of left circumflex (LCX); 13. distal segment of LCX; (14) first obtuse marginal branch; (15) second obtuse marginal branch; (16) left posterolateral artery. In this example, plaque distribute on proximal segment of RCA, proximal segment of LAD and proximal segment of LCX respectively. Segment involvement score was calculated by summation of the involved number of coronary segments. The segment involvement score in this example is 3 out of a possible 16. Segment stenosis score was calculated by summation of minimal plaque in the proximal RCA (scored 1), mild plaque in the proximal LAD (scored 2) and severe plaque in the proximal LCX (scored 4).The segment stenosis score is 7 out of a possible 80

**Fig. 2:**
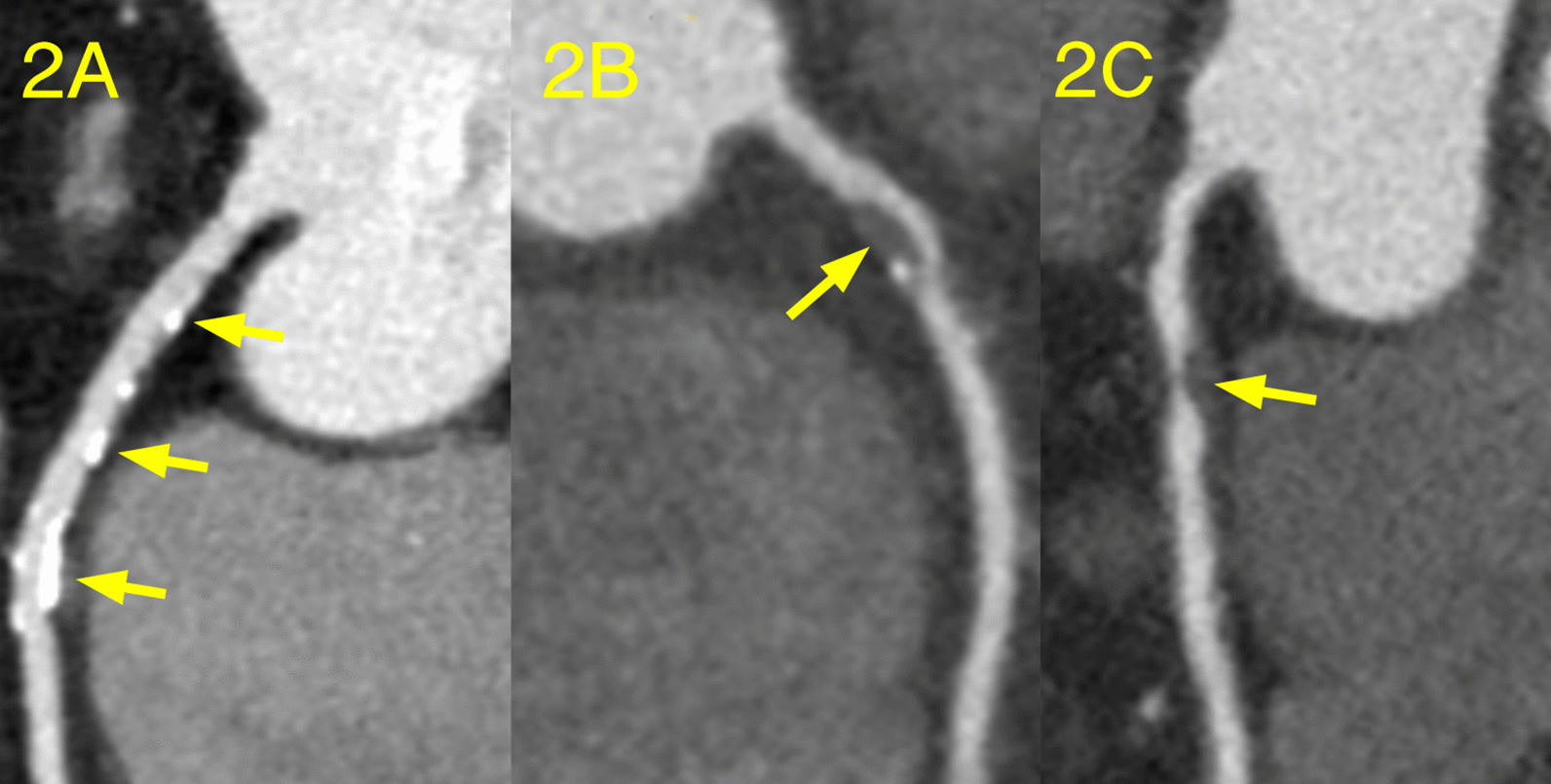
Type of plaques and grade of lumen stenosis **a** Multiple calcified plaques, causing minimal to mild stenosis of vascular lumen. **b** Mixed plaque, causing in moderate stenosis of vascular lumen. **c** Non-calcified plaque, causing severe stenosis of vascular lumen

Two cardiovascular radiologists with at least three years of diagnosis experience independently analyzed the images. Discrepancies of two observers in interpretations were resolved by consensus. In this study, coronary arteries were divided into four branches and 16 separate segments based on a modified AHA classification [15] (Fig. 1). Plaques were classified as calcified plaque (plaques with higher CT density than contrast-enhanced lumen); non- calcified plaque (plaques with lower CT attenuation than contrast-enhanced lumen without any calcification) and mixed plaque (non-calcified and calcified elements in single plaque) [16]. For each coronary artery plaque detected, lumen stenosis were graded as a 5-point scale based on CAD-RADS [17]: Grade 0:no visible stenosis; Grade 1-minimal (1–24% luminal stenosis); Grade 2-mild (25–49% luminal stenosis); Grade 3-moderate (50–69% luminal stenosis); Grade 4-severe (70–99% luminal stenosis); Grade 5-totally occluded (Fig. 2). Obstructive stenosis is defined as luminal stenosis ≥ 50%. The number of various types of plaques and different grades of stenosis in each patient were counted. Finally, plaque burden was assessed on a per-patient basis using previous validated CT scores included Segment involvement scores (SIS), which represents the total number of coronary artery segments with plaques (range 0–16) and Segment stenosis scores (SSS), which calculated as the summation of the stenosis scores of all 16 individual segments (range 0–80) [18, 19].

### Statistical analysis

Statistical analysis was performed using SPSS software (version 23.0). Baseline clinical and laboratory data, number of involved coronary vessels and segments, number and types of plaques, extent of luminal narrowing caused by plaques, SIS and SSS were statistically evaluated for each patient. Categorical variables were expressed as the number (%) and compared using chi-square test. Continuous variables were expressed as the mean ± standard deviation. For the comparison of different grades of SCH, one-way analysis of variance was used for the variables with normal distributed and homogeneous variance. Kruskal–Wallis rank test was used was used for the variables with non-normally distributed or uneven variance, with post hoc pairwise comparisons with Bonferroni correction. For the sex comparisons, an unpaired Student *t*-test was used for normally distributed variables, and Mann–Whitney *U* tests were used for non-normally distributed variables. Logistic regression analysis was used to analyze the risk factors such as age, sex, BMI and other clinical variables for non-calcified plaque. Linear regression analysis was used to analyze above risk factors on segment stenosis scores scores (SSS). Two-tailed *p* values < 0.05 were considered statistically significant.

## Results

### Clinical characteristics and laboratory data of the patients, stratified by grades of SCH and sex

The patients’ baseline clinical and laboratory data are shown in Tables 1 and 2. The serum FT4 level of patients with mild SCH was higher than in the other two groups, and the serum FT4 level of patients with moderate SCH was higher than in those with severe SCH (both adjusted *p* < 0.05). In addition, there were no significant discrepancies among the three groups in age, sex, body mass index, other high-risk factors for CAD, or the use of statins or antiplatelets. Among the different sex, a higher proportion of men were found to be smokers as compared with women (23.1% vs. 2.0%, *p* < 0.001). There were no significant differences in other high-risk factors of CAD and grades of SCH between the different sex.

**Table 1:** Baseline clinical and laboratory characteristics of different grades of SCH group

Characteristics	SCH patients (n = 234)	Mild SCH (n = 143)	Moderate SCH (n = 62)	Severe SCH (n = 28)	*p* value
Age (year)	71.5 ± 9.8	72.0 ± 9.8	70.9 ± 9.4	69.9 ± 10.2	0.514
Male (%)	116 (49.6)	75 (52.4)	27 (43.5)	14 (50)	0.456
BMI (kg/m^2^)	23.6 ± 4.6	23.9 ± 3.1	22.8 ± 5.2	24.2 ± 8.2	0.273
Hypertension (%)	152 (65)	93 (65)	43 (69.4)	16 (57.1)	0.591
SBP (mmHg)	136.7 ± 20.3	137.7 ± 19.8	136.2 ± 21.8	132.6 ± 20.1	0.518
DBP (mmHg)	79.2 ± 13.5	79.1 ± 13.4	80.3 ± 13.3	77.4 ± 14.9	0.681
DM (%)	79 (33.8)	56 (39.2)	16 (25.8)	7 (25)	0.091
Duration of DM (year)	3.5 ± 6.9	4.1 ± 7.6	2.5 ± 5.3	2.8 ± 6.2	0.271
Hyperlipemia (%)	47 (20)	27 (18.9)	12 (19.4)	8 (28.5)	0.660
Smoking (%)	33 (14.1)	22 (15.4)	6 (9.7)	2 (7)	0.601
Using of statins (%)	39 (16.7)	28 (20.9)	7 (11.3)	4 (14.3)	0.293
Using of antiplatelet agents (%)	39 (16.7)	25 (17.5)	10 (16.1)	4 (14.3)	0.903
Fasting blood-glucose (mmol/L)	6.4 ± 2.3	6.5 ± 2.3	6.2 ± 2.4	6.4 ± 2.1	0.730
FT3 (pmol/L)	4.5 ± 2.5	4.7 ± 3.2	4.4 ± 0.8	4.1 ± 0.8	0.510
FT4 (pmol/L)	15.7 ± 2.1	16.0 ± 2.1	15.3 ± 2.1*	14.7 ± 2.0*^#^	< 0.001
TSH (mU/L)	7.2 ± 3.1	5.5 ± 0.7	8.0 ± 0.8*	13.7 ± 4.2*^#^	< 0.001
TG (mmol/L)	1.5 ± 0.9	1.6 ± 0.9	1.5 ± 0.8	1.5 ± 0.7	0.932
TC (mmol/L)	4.0 ± 1.0	3.9 ± 1.0	4.1 ± 1.2	4.2 ± 1.3	0.354
HDL-C (mmol/L)	1.3 ± 0.4	1.2 ± 0.4	1.3 ± 0.5	1.3 ± 0.5	0.345
LDL-C (mmol/L)	2.2 ± 0.8	2.2 ± 0.8	2.2 ± 0.9	2.4 ± 0.9	0.519

*Adjusted *p* < 0.05 versus Mild SCH;#adjusted *p* < 0.05 versus Moderate SCH. BMI, body mass index; DM, diabetes mellitus; FT3, free triiodothyronine; FT4, free thyroxine; TG, triglycerides; Cho, cholesterol; HDL-C, high-density lipoprotein cholesterol; LDL-C, low-density lipoprotein cholesterol

**Table 2:** Baseline clinical and laboratory characteristics of different sexes group

	Men with SCH (n = 116)	Women with SCH (n = 118	P值
Age (year)	71.0 ± 10.6	71.9 ± 8.9	0.472
BMI (kg/m^2^)	24.1 ± 5.2	23.1 ± 3.9	0.122
Hypertension (%)	80 (68.9)	72 (61.0)	0.220
SBP (mmHg)	136.4 ± 22.5	136.7 ± 17.4	0.913
DBP (mmHg)	79.8 ± 14.6	78.0 ± 11.6	0.290
DM (%)	39 (33.6)	40 (33.9)	0.964
Duration of DM (year)	3.4 ± 6.6	3.5 ± 7.2	0.883
Hyperlipemia (%)	21 (18.1)	26 (22.0)	0.397
Smoking (%)	24 (23.1)	2 (2.0)	< 0.001
Using of statins (%)	17 (14.7)	22 (18.6)	0.484
Using of antiplatelet agents (%)	23 (19.8)	16 (13.6)	0.224
Fasting blood-glucose (mmol/L)	6.6 ± 2.5	6.2 ± 2.1	0.235
Mild SCH (%)	75 (64.7)	68 (57.6)	0.378
Moderate SCH (%)	27 (23.3)	36 (30.5)	0.291
Severe SCH (%)	14 (12.1)	14 (11.9)	0.814
FT3 (pmol/L)	4.8 ± 1.6	4.2 ± 0.7	0.082
FT4 (pmol/L)	15.8 ± 2.0	15.6 ± 2.3	0.444
TSH (mU/L)	7.0 ± 2.8	7.4 ± 3.3	0.332
TG (mmol/L)	1.5 ± 0.7	1.6 ± 1.0	0.136
TC (mmol/L)	3.9 ± 1.0	4.2 ± 1.1	0.054
HDL-C (mmol/L)	1.2 ± 0.4	1.3 ± 0.4	0.079
LDL-C (mmol/L)	2.1 ± 0.8	2.3 ± 0.9	0.164

### Plaque distribution and coronary artery stenosis in different grades of SCH

**Fig. 3:**
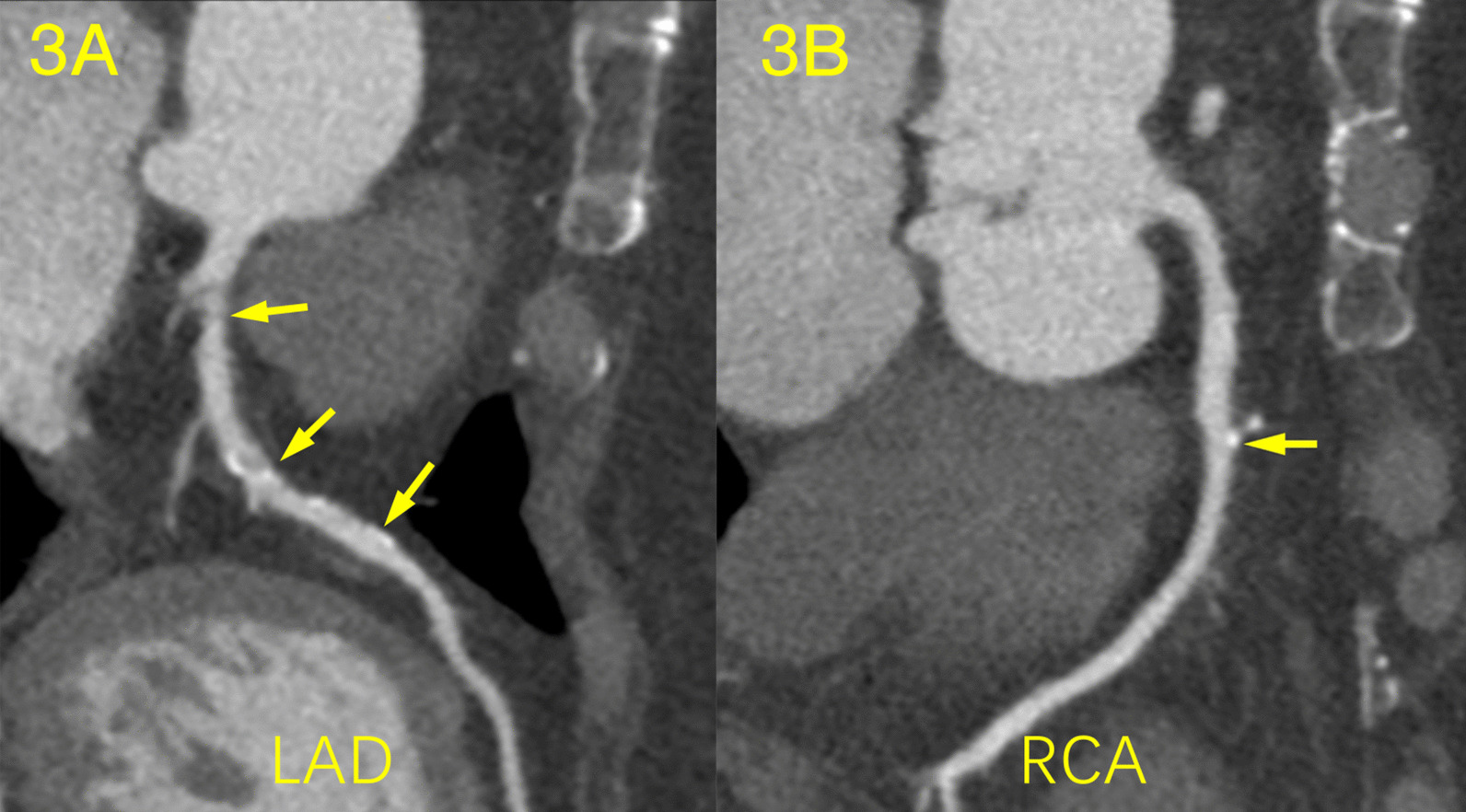
Calcified and mixed plaque in a 71-year-old woman with mild SCH and DM. The patient underwent coronary CTA for palpitations. **a** Curvature planar reconstruction CT image of the LAD artery shows several mixed plaques in the proximal and middle segments of the LAD with mild stenosis. **b** The image of the RCA artery shows small calcified plaque in the middle segment of the RCA with minimal stenosis

**Fig. 4:**
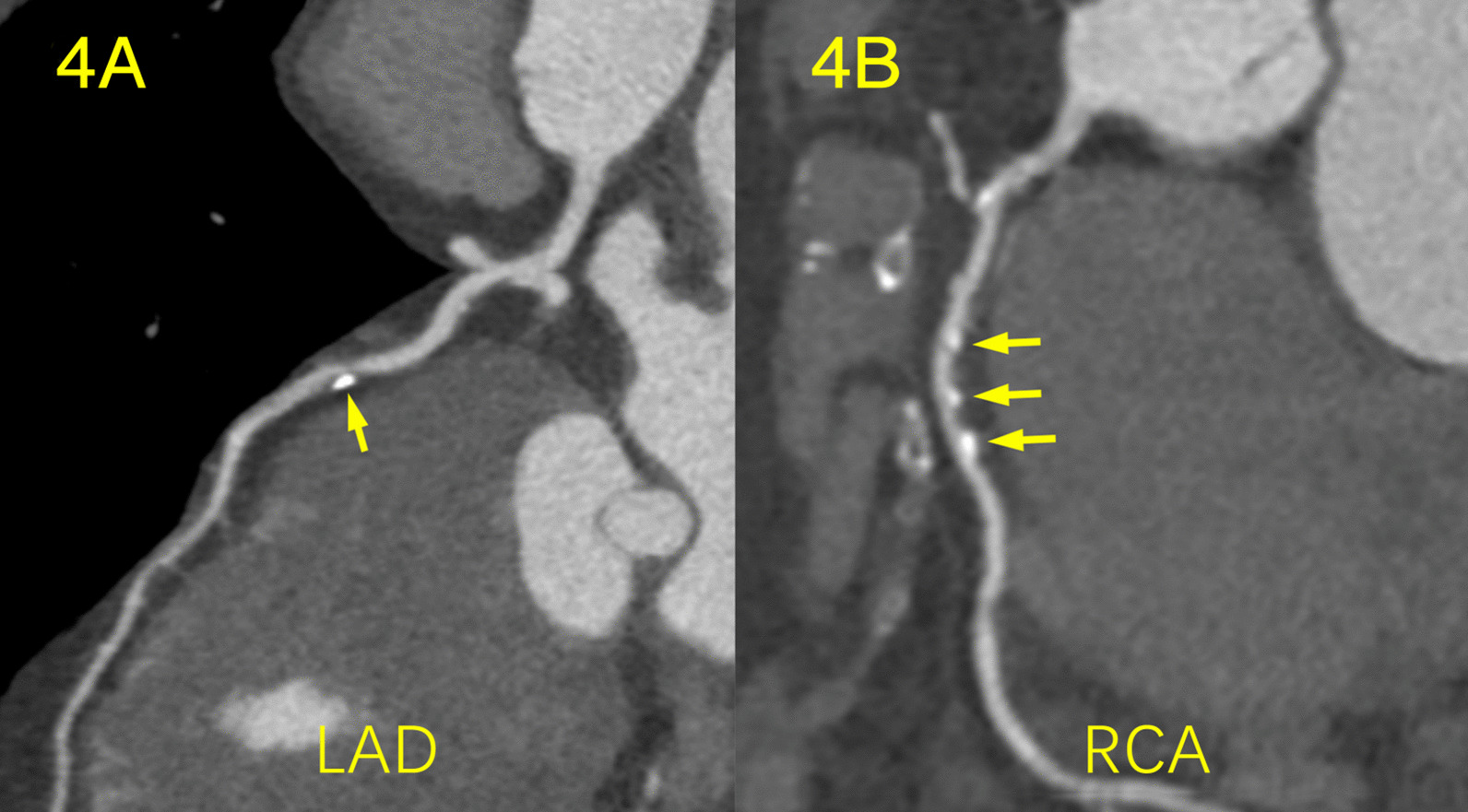
Mixed plaque in a 55-year-old man with moderate SCH and hypertension. The patient underwent coronary CTA for chest tightness. **a** Curvature planar reconstruction CT image of the LAD artery shows a mixed plaque in the proximal segment of the LAD with moderate stenosis. **b** The image of the RCA artery shows a long mixed plaque in proximal and middle segments of the RCA with moderate stenosis

**Fig. 5:**
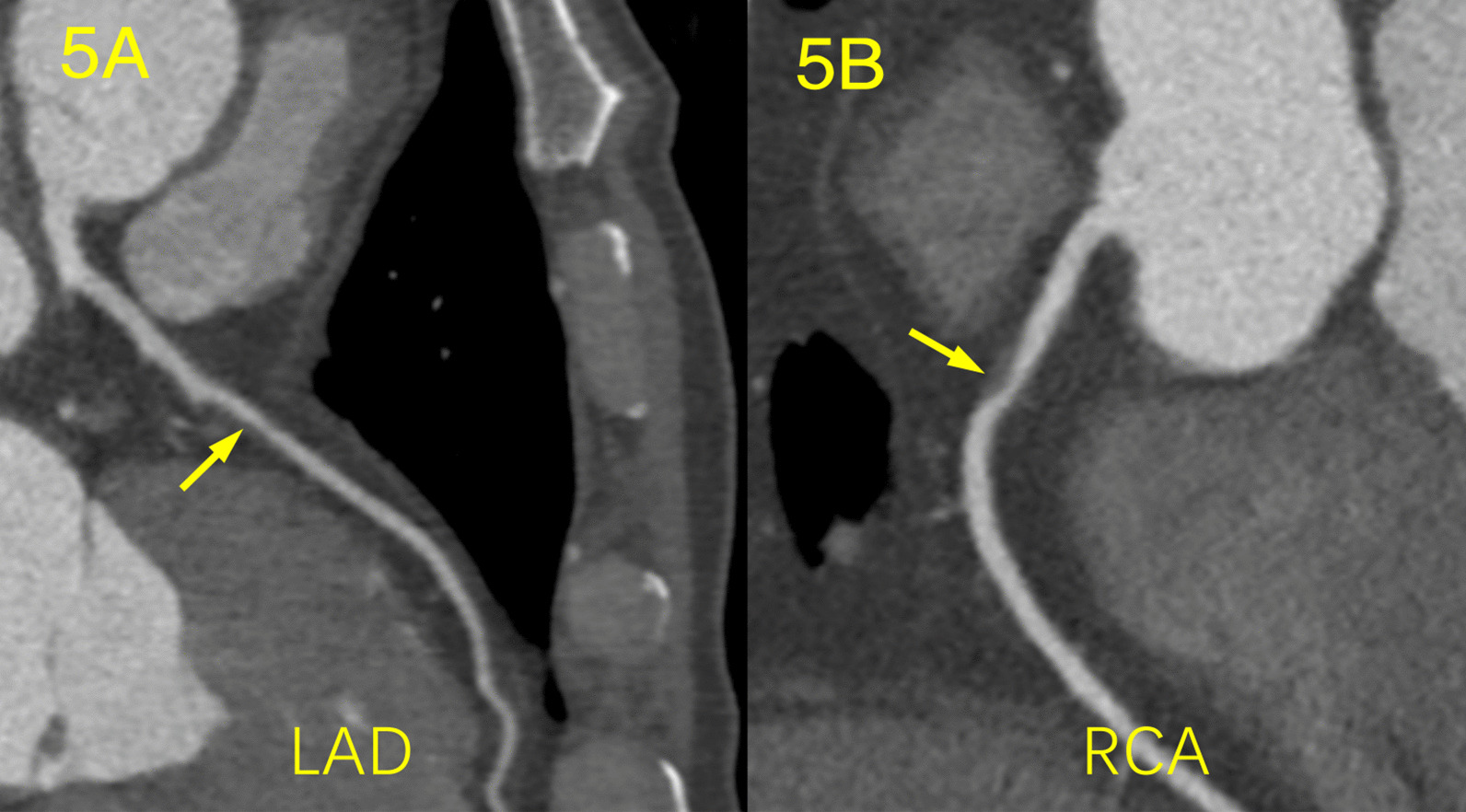
Non-calcified plaques in a 59-year-old man with severe SCH. The patient underwent coronary CTA for chest pain. **a** Curvature planar reconstruction CT image of the LAD artery shows a non-calcified plaque in the middle segment of the LAD with mild stenosis. **b** The image of the RCA artery shows a non-calcified plaque in proximal segment of the RCA with severe stenosis

**Fig. 6:**
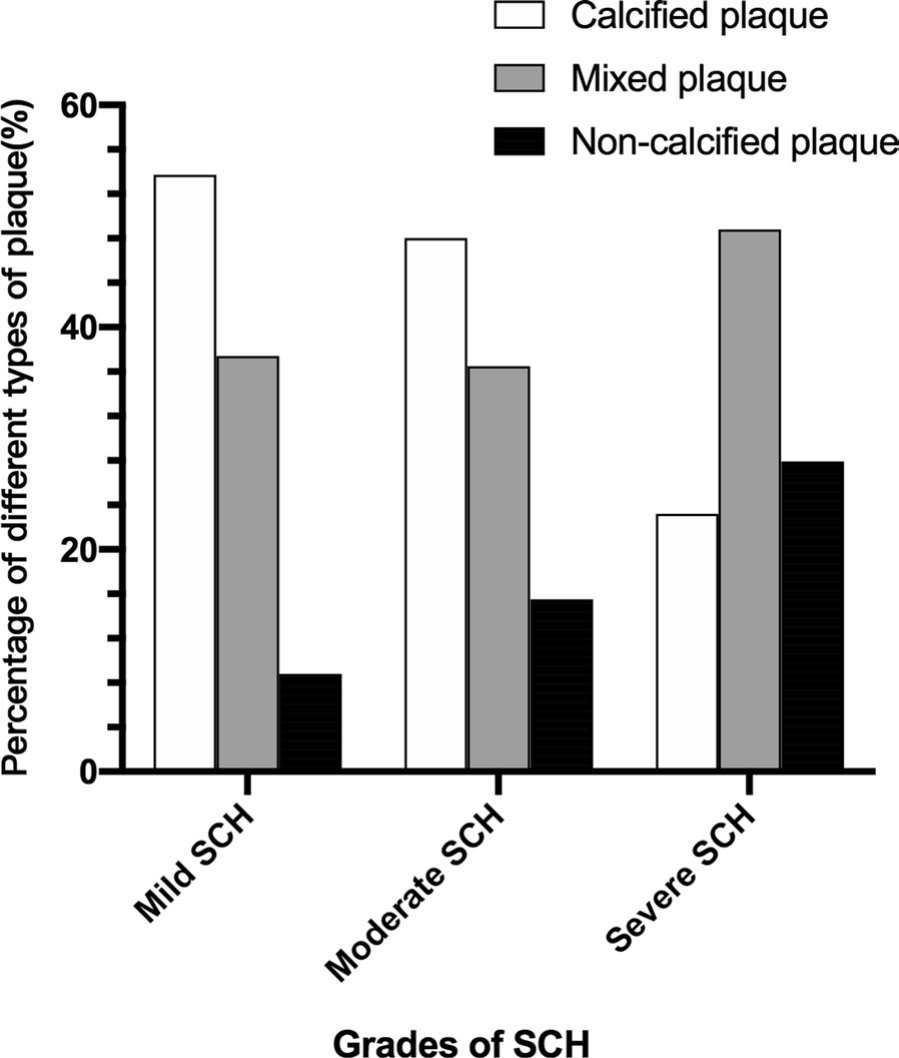
The graph shows the percentage of different types of plaques in different grades of SCH groups. The proportion of non-calcified plaques increases as the grade of SCH increases. And the proportion of calcified plaques in severe SCH group is lower than mild and moderate SCH groups

A total of 807 plaques and 784 stenoses were detected, involved 503 vessels and 805 segments of coronary arteries. The plaque distribution and coronary artery stenosis of the different grades of SCH are shown in Table 3. Patients with mild SCH had a greater number of calcified plaques than those with severe SCH (2.0 ± 1.9 vs. 0.7 ± 0.9, *p* < 0.001). There was no significant difference in the number of mixed plaques among the patients with different grades of SCH, whereas the severe SCH group had more non-calcified plaques than the mild SCH group (0.9 ± 1.0 vs. 0.3 ± 0.5, *p* < 0.001) (Figs. 3, 4, 5). As the SCH condition worsened, the proportion of non-calcified plaques significantly increased (Fig. 6). Patients with severe SCH had more mild stenosis than the other two groups (*p* < 0.05). In addition, there was no significant difference in SIS or SSS among different degree of SCH groups.

**Table 3:** Comparison of coronary artery disease between different grades of SCH

	All patients (n = 234)	Mild SCH (n = 143)	Moderate SCH (n = 62)	Severe SCH (n = 28)	*p* value
Types of plaque					
Calcified plaque	1.7 ± 1.8	2.0 ± 1.9	1.4 ± 1.6	0.7 ± 0.9*	< 0.001
Mixed plaque	1.3 ± 1.6	1.4 ± 1.9	1.1 ± 1.6	1.5 ± 1.7	0.302
Non-calcified plaque	0.4 ± 0.7	0.3 ± 0.5	0.5 ± 0.7	0.9 ± 1.0*	< 0.001
Grading of stenosis					
Minimal stenosis	1.1 ± 1.3	1.2 ± 1.4	1.2 ± 1.5	0.5 ± 0.8*^#^	0.021
Mild stenosis	1.4 ± 1.4	1.4 ± 1.5	1.2 ± 1.4	1.8 ± 1.2*^#^	0.001
Moderate stenosis	0.6 ± 0.7	0.6 ± 1.0	0.5 ± 0.9	0.6 ± 0.8	0.848
Severe stenosis	0.2 ± 0.5	0.2 ± 0.5	0.2 ± 0.6	0.1 ± 0.2	0.069
Obstructive stenosis	0.4 ± 0.4	0.4 ± 0.5	0.4 ± 0.5	0.5 ± 0.5	0.831
SIS	3.5 ± 2.4	3.7 ± 2.4	2.9 ± 2.4	3.0 ± 1.8	0.084
SSS	6.8 ± 6.0	7.4 ± 6.4	5.4 ± 5.6	6.0 ± 3.5	0.092

*Adjusted *p* < 0.05 versus Mild SCH;#adjusted *p* < 0.05 versus Moderate SCH.SIS, segment involvement scores; SSS, segment stenosis scores

### Comparison of characteristics of CAD between different sexes

**Fig. 7:**
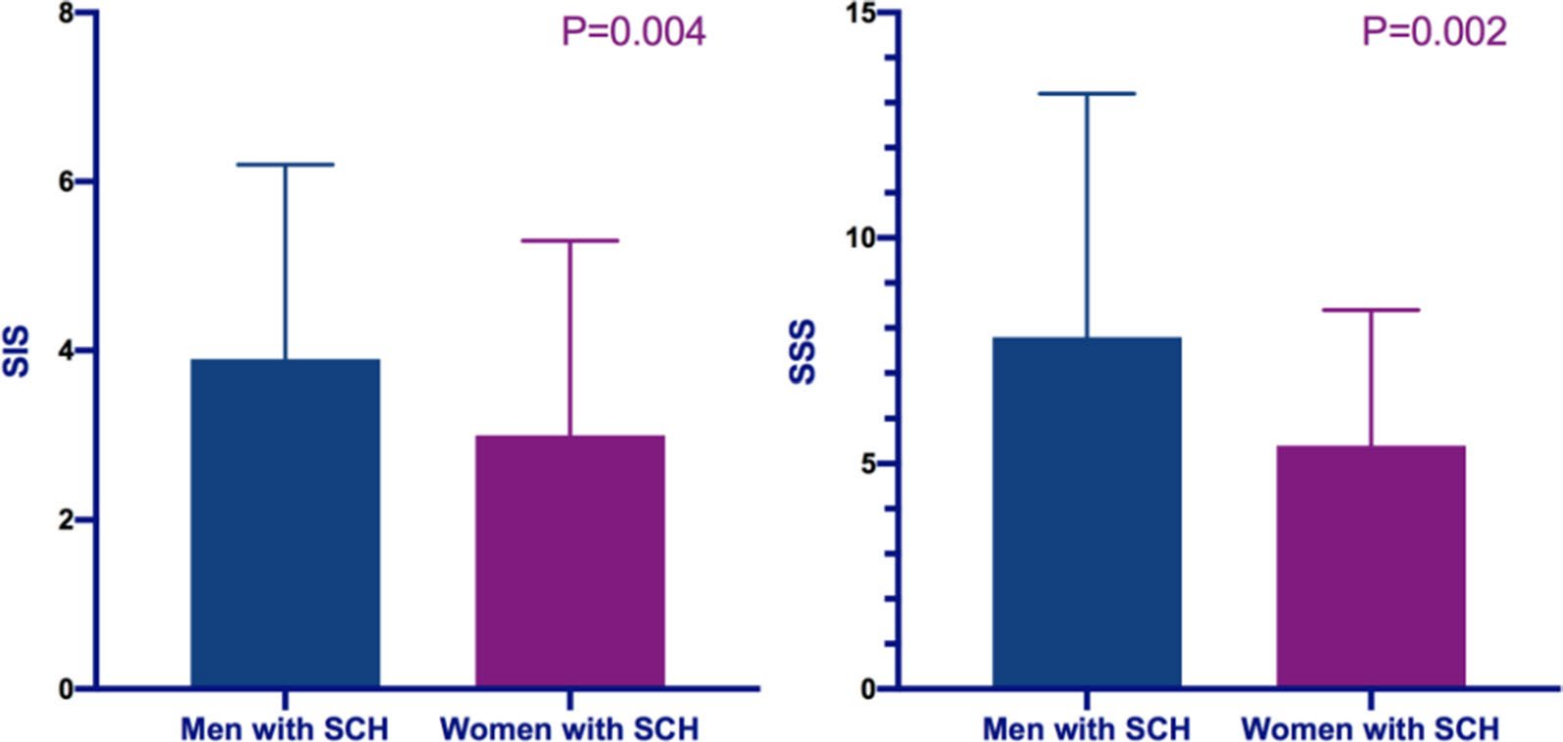
The graph shows the comparison of SIS and SSS scores between different sexes groups

Table 4 shows the comparison of type of plaques, severity of stenosis, and extent of CAD between men and women with SCH. In terms of types of coronary plaques, men had more mixed plaques than women (1.7 ± 2.0 vs. 1.0 ± 1.4, *p* = 0.002), and there was no significant difference in the number of other types of plaques between different sex groups. Men had more moderate stenosis than women (*p* < 0.05). In addition, men had higher SIS (3.9 ± 2.3 vs. 3.0 ± 2.3, *p* = 0.004) and SSS (7.8 ± 5.4 vs. 5.4 ± 3.0, *p* = 0.002) than women (Fig. 7).

**Table 4:** Comparison of coronary artery disease between different sexes

	Men with SCH (n = 116)	Women with SCH (n = 118)	*p* value
Types of plaque			
Calcified plaque	1.7 ± 1.8	1.7 ± 1.9	0.704
Mixed plaque	1.7 ± 2.0	1.0 ± 1.4	0.002
Non-calcified plaque	0.5 ± 0.8	0.4 ± 0.8	0.363
Grading of stenosis			
Minimal stenosis	1.2 ± 1.3	1.1 ± 1.3	0.560
Mild stenosis	1.6 ± 1.6	1.3 ± 1.3	0.165
Moderate stenosis	0.8 ± 1.0	0.4 ± 0.8	0.003
Severe stenosis	0.2 ± 0.6	0.2 ± 0.4	0.644
Obstructive stenosis	0.5 ± 0.5	0.4 ± 0.5	0.171
SIS	3.9 ± 2.3	3.0 ± 2.3	0.004
SSS	7.8 ± 5.4	5.4 ± 3.0	0.002

### Univariate and multivariate analysis of risk factors of non-calcified plaques and SSS in SCH patients

The results of the univariate and multivariate analysis of risk factors of presence of non-calcified plaques, and risk factors of SSS are shown in Tables 5 and 6, respectively. Multivariate analysis showed that after adjusting for confounding factors, grades of SCH (Moderate SCH, odds ratio [OR]: 2.11; 95% CI 1.03–4.34, *p* = 0.042; severe SCH, OR: 10.00; 95% CI 3.82–26.20, *p* < 0.001, taken mild SCH as a reference), and smoking (OR: 3.06; 95% CI 1.34–6.99, *p* = 0.008) were independently associated with the prevalence of non-calcified plaques. Sex (B: 1.67; 95% CI 0.27–3.10, *p* = 0.009) and DM (B: 2.23; 95% CI 0.68–3.77, *p* = 0.005) were independently associated with SSS.

**Table 5:** Independent predictors for prevalence of non-calcified plaques

	Univariate		Multivariate	
	OR (95% CI)	*p* value	OR (95% CI)	*p* value
Sex	1.26 (0.72–2.19)	0.426	–	0.872
Age	0.97 (0.94–1.00)	0.026	–	0.245
BMI	1.00 (0.92–1.05)	0.644	–	0.787
DM	1.43 (0.80–2.55)	0.227	–	0.235
Hypertension	0.70 (0.39–1.24)	0.221	–	0.405
Hyperlipemia	1.15 (0.54–2.31)	0.561	–	0.964
Using of statins	0.89 (0.41–1.90)	0.751	–	0.683
Using of antiplatelet agents	1.02 (0.48–2.14)	0.965	–	0.837
Smoking	2.50 (1.18–5.29)	0.017	3.06 (1.34–6.99)	0.008
Grade of SCH				
Mild	Reference	–	Reference	–
Moderate	1.73 (0.90–3.34)	0.099	2.11 (1.03–4.34)	0.042
Severe	6.24 (2.62–14.86)	< 0.001	10.00 (3.82–26.20)	< 0.001

**Table 6:** Independent risk factors for segment involvement scores (SSS)

	Univariate		Multivariate		
	Β (95% CI)	*p* value	Β (95% CI)	β	*p* value
Sex	2.54 (0.92–4.16)	0.001	1.67 (0.27–3.10)	0.16	0.009
Age	0.05 (− 0.04 to 0.13)	0.264	–	–	0.948
BMI	0.08 (− 0.08 to 0.25)	0.336	–	–	0.945
DM	2.72 (1.15–4.30)	0.001	2.23 (0.68–3.77)	0.19	0.005
Hypertension	2.21 (0.63–3.78)	0.006	–	–	0.073
Hyperlipemia	0.57 (-1.32–2.47)	0.552	–	–	0.923
Using of statins	0.59 (− 1.46 to 2.63)	0.574	–	–	0.438
Using of antiplatelet agents	1.12 (− 0.93 to 3.12)	0.281	–	–	0.239
Smoking	0.27 (− 1.92 to 2.46)	0.807	–	–	0.855
Grade of SCH					0.940
Mild	Reference	–	Reference	–	–
Moderate	− 2.15 (− 3.90 to 0.12)	0.016	–	–	0.541
Severe	− 1.28 (− 3.66 to 1.11)	0.292	–	–	0.819

## Discussion

Our study has three main findings. First, as compared with mild SCH patients, patient with severe SCH had fewer calcified plaques and more non-calcified plaques. As the condition of SCH worsened, the proportion of non-calcified plaques increased. Second, men with SCH had a higher plaque burden than women with SCH. Third, grades of SCH, especially severe SCH, was associated with the prevalence of non-calcified plaques, and in our research population, the total plaque burden was independently associated with sex.

Coronary CTA has a high sensitivity in the detection of small plaque [20]. Most of the patients in our study underwent coronary CTA examination because of related clinical symptoms. The majority of patients were found to have coronary plaque. This may be due to the advantage of coronary CTA in displaying small lesions.

Traditional examinations for CAD, such as coronary angiography, have a greater focus on the assessment of lumen stenosis. However, plaque characteristics are equally as important as the degree of stenosis in the assessment of risk and prognosis of patients with CAD. Previous studies have confirmed the role of coronary CTA in the evaluation of the components of atherosclerosis plaques, even beyond the quantification of lumen stenosis [21, 22]. Non-calcified plaques are usually characterized as lipid-rich and are generally considered to be more unstable than other plaque types. Elevated non-calcified plaque volume may increase the incidence of acute coronary syndrome, cardiac death, and major adverse cardiovascular events [16, 23].

SCH can accelerate the inflammatory response, lead to disorders of lipid metabolism, and aggravate atherosclerosis [24–26]. Insulin resistance can increase the risk of coronary artery restenosis after percutaneous coronary intervention, while adiponectin has a protective effect on restenosis [27]. Moreover, serum adiponectin level is negatively correlated with TSH level [28]. It has been shown that SCH is an independent risk factor for CAD [9, 29, 30]; severe SCH is associated with increased CAD mortality, stroke, and heart failure [31, 32]. In our study, patients with severe SCH had fewer calcified plaques and more non-calcified plaques than patients with mild SCH. Severe SCH was an independent risk factor for the prevalence of non-calcified plaques. SCH may aggravate inflammatory activity and make atherosclerotic plaque unstable. While L-thyroxine treatment might contribute to plaque stabilization by inhibiting the innate immunity-dependent plaque rupture [33]. A study using optical coherence tomography revealed that patients with SCH had more lipid-rich plaques and larger lipid arcs than patients without SCH, although the results were limited because of the small sample size [34]. Taken together, the result of our study and those of previous research demonstrate that the degree of thyroid function failure may correlate with an increase in non-calcified plaques. This may explain why severe SCH is related to higher CAD mortality and worse long-term prognosis. However, although patients with severe SCH had more mild stenosis than those with other grades of SCH, overall plaque burden seemed unaffected by SCH grade in our study. This may be because the percentage of DM and smoking was higher in the mild SCH group than severe SCH group. Although the difference was not statistically significant, it may have influenced the results.

Hypothyroidism leads to atherosclerosis through multiple mechanisms, with dyslipidemia playing a crucial role in its pathophysiology. TSH can upregulate hepatic 3-hydroxy-3-methyl-glutaryl coenzyme A reductase, resulting in hypercholesterolemia [35]. Studies have confirmed that total serum cholesterol, low-density lipoprotein cholesterol, and total triglyceride levels were significantly increased in patients with SCH when compared with euthyroid individuals [36, 37]. In addition, elevated lipid levels could be improved after L-thyroxin replacement therapy in patients with subclinical and overt hypothyroidism [38]. In our study, there was no significant difference was observed in lipid profiles of patients with different grades of SCH. This difference might have been due to the different study populations.

In addition to dyslipidemia, inflammation is another mechanism of SCH leading to atherosclerosis. TSH could directly bind to TSH receptors in macrophages, thereby aggravating vascular inflammation and contributing to atherogenesis [39]. Another previous study showed that the serum TSH level was positively associated with circulating retinol-binding protein 4 (RBP4) [40]. RBP4 could contribute to insulin resistance, and high levels of circulating RBP4 are associated with atherosclerosis and CAD [41]. Different plaque distributions in different grades of SCH may be associated with serum TSH levels in addition to dyslipidemia.

In our study, overall plaque burden seemed unaffected by SCH grade. However, differences in sex affected total plaque burden. In patients with SCH, men had higher SIS and SSS than women. Although the SIS and SSS score systems have limitations in providing further information such as plaque localization, they are effective for describing total plaque burden [16, 42, 43]. In our study, the proportion of smokers was higher in men than women in our study, whereas smoking was a confounding factor for CAD. However, after correcting for confounding factors, sex was found to be independently associated with SSS, while smoking was not associated with SSS. A study demonstrated that patients with SCH who were at an intermediate-to-high risk of CAD, especially men with SCH, were significantly more likely to develop CAD [44]. Furthermore, SCH might be a risk factor for cardiovascular disease in men who are less than 50 years old [45].

The difference in CAD between men and women with SCH, may be related to impaired endothelial function. A previous study demonstrated that elevated serum TSH levels were more significantly associated with impaired endothelial function in men than in women [7]. This may be because elevated serum TSH levels could impair endothelial function via the NO system, and endothelial function is more sensitive in men than in women [46, 47].

It should be noted that most of the patients in our study are elderly patients. The elderly are relatively fragile subjects [48]. There is a gender dimorphism in the demographic and morbidity profiles of hospitalized elderly people [49]. Also, SCH has gender differences in the incidence rate and comorbidities. Our research indicates that elderly men with SCH have higher coronary plaque burden than women. It is necessary to carry out personalized process of health care for patients according to age, gender, and other factors.

Our study has some limitations. First, this was a single-center study, and a possible selection bias cannot be ignored. Second, due to the retrospective nature of this study, our results need to be confirmed by a prospective cohort study. Thirdly, we did not systematically compare our findings on coronary artery CTA with coronary angiography for luminal stenosis assessments, as the high diagnostic accuracy of coronary artery CTA for the assessment of CAD is widely accepted. Fourth, because our study subjects were mainly the elderly people. Therefore, whether the results can be extended to young people needs to be confirmed by future studies.

## Conclusions

Grades of SCH, especially severe SCH, is associated with coronary non-calcified plaques, which is an unstable and vulnerable type of plaque that leads to major adverse cardiovascular events. This finding suggests that it is important to evaluate for coronary plaque in SCH patients, especially in those with severe SCH. In addition, among SCH patients, men had a higher total plaque burden than women. Further studies are warranted to confirm the sex difference in CAD among patients with SCH.

## Data Availability

The datasets used and/or analyzed during the current study are available from the corresponding author on reasonable request.

## Abbreviations

SCH: Subclinical hypothyroidism
CAD: Coronary artery disease
CCTA: Coronary computed tomographic angiography
SIS: Segment involvement score
SSS: Segment stenosis score
OR: Odds ratio
DM: Diabetes mellitus
TSH: Thyroid-stimulating hormone
FT4: Free thyroxine
RBP4: Retinol-binding protein 4
BMI: Body mass index
FT3: Free triiodothyronine
TG: Triglycerides
Cho: Cholesterol
HDL-C: High-density lipoprotein cholesterol
LDL-C: Low-density lipoprotein cholesterol
